# Supplementary figures and images for: Evaluation of Reference Gene Stability in Goat Skeletal Muscle Satellite Cells during Proliferation and Differentiation Phases
Source: Animals (Basel). 2024 Aug 26;14(17):2479. doi: 10.3390/ani14172479 (PMC11394193; doi:10.3390/ani14172479)

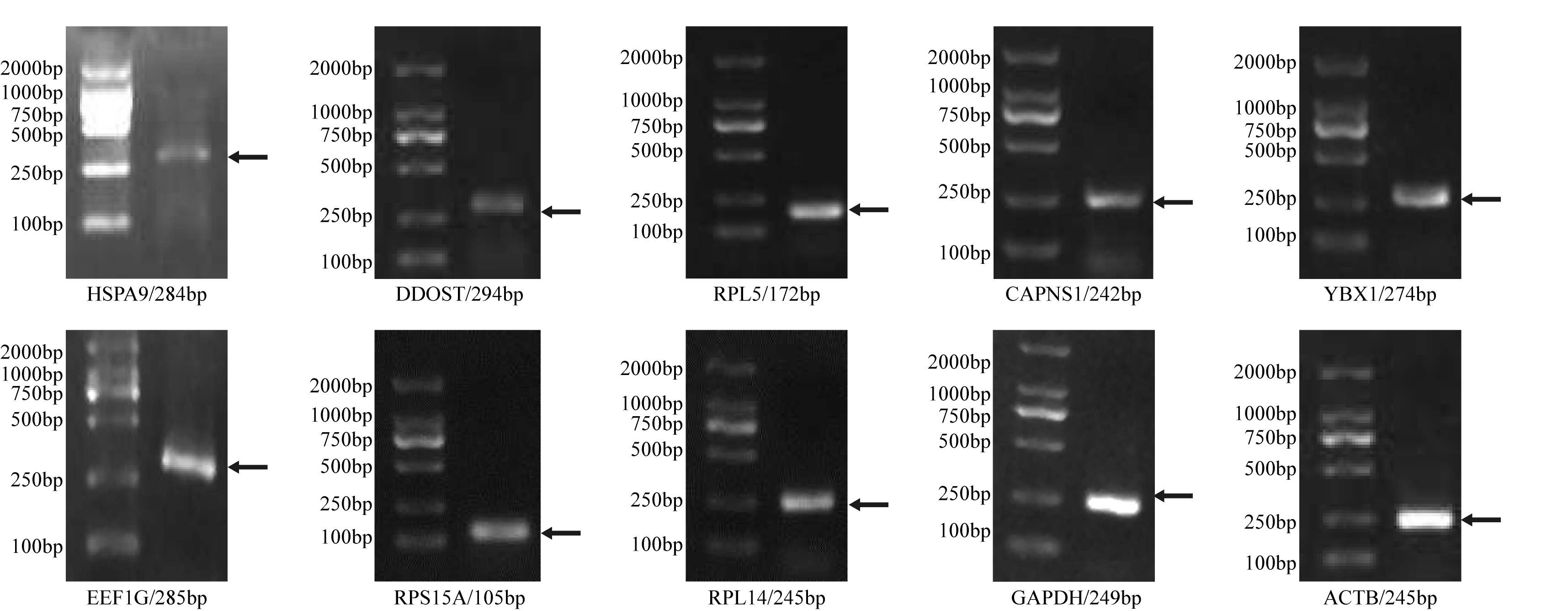

Supplement: Supplementary file 1 [file animals-14-02479-s001.zip › Supplementary Figure S1.tif]

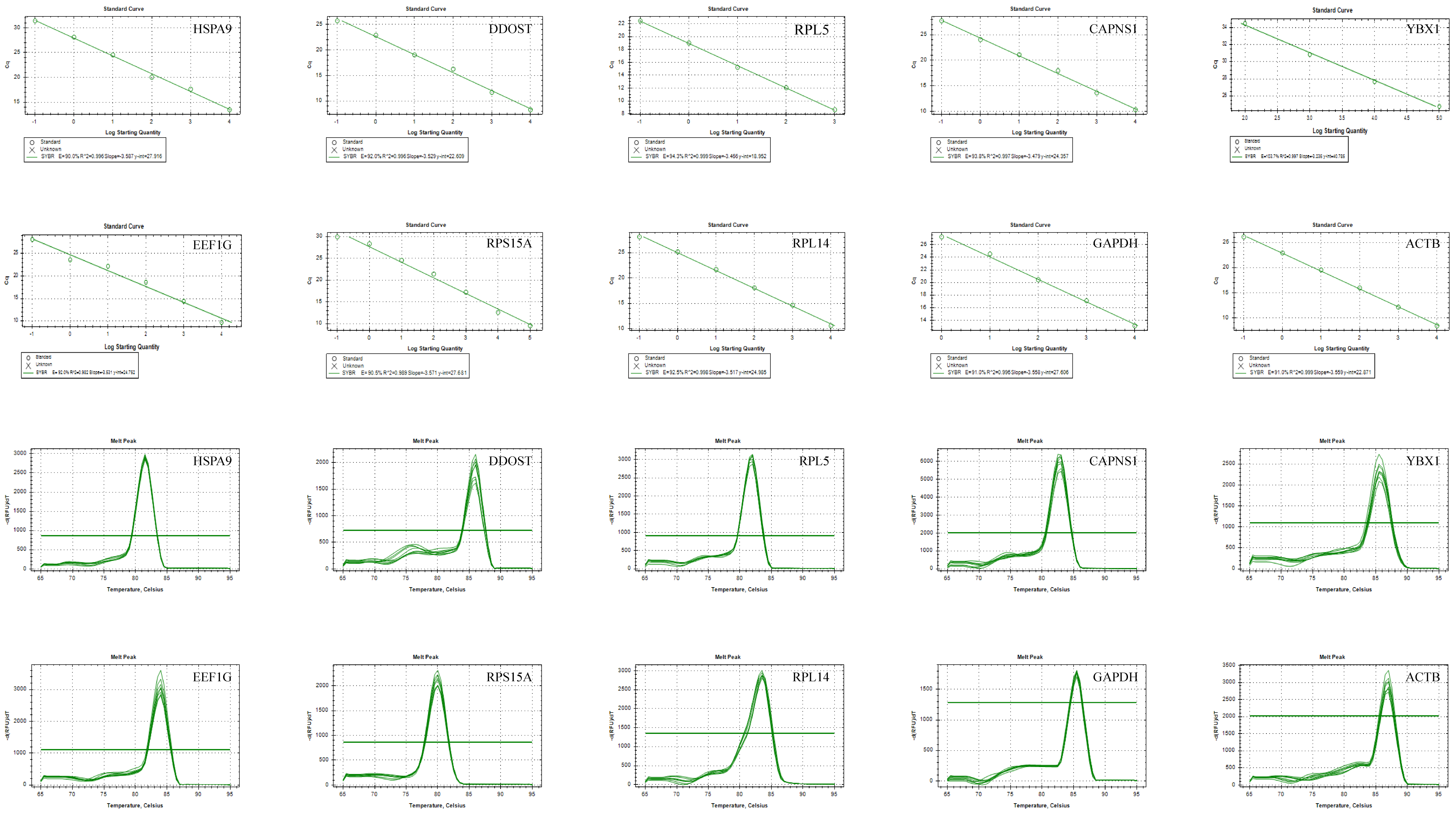

Supplement: Supplementary file 1 [file animals-14-02479-s001.zip › Supplementary Figure S2.tif]
